# Crystal structure and Hirshfeld surface analysis of 3-methyl-4-oxo-*N*-phenyl-3,4-di­hydro­quinazoline-2-carbo­thio­amide

**DOI:** 10.1107/S2056989021013116

**Published:** 2022-01-01

**Authors:** Nasiba Pirnazarova, Ubaydullo Yakubov, Sevara Allabergenova, Akmaljon Tojiboev, Kambarali Turgunov, Burkhon Elmuradov

**Affiliations:** a Qarshi State University, Kochabog str. 17, Qarshi 180119, Uzbekistan; b S. Yunusov Institute of Chemistry of Plant Substances, Academy of Sciences of Uzbekistan, Mirzo Ulugbek str. 77, Tashkent 100170, Uzbekistan; c University of Geological Sciences, Olimlar str. 64, Mirzo Ulugbek district, Tashkent, Uzbekistan; d National University of Uzbekistan named after Mirzo Ulugbek 100174, University Str. 4, Olmazor District, Tashkent, Uzbekistan; e Turin Polytechnic University in Tashkent, Kichik Khalka yuli str. 17, 100095 Tashkent, Uzbekistan

**Keywords:** crystal structure, thio­amide, intra- and inter­molecular inter­actions, Hirshfeld surface

## Abstract

The asymmetric unit of the title compound, C_16_H_13_N_3_OS, comprises two mol­ecules (*A* and *B*) with similar conformations that differ mainly in the orientation of the phenyl group relative to the rest of the mol­ecule, as expressed by the C_thio­amide_—N_thio­amide_—C_phen­yl_—C_phen­yl_ torsion angle of 49.3 (3)° for mol­ecule *A* and of 5.4 (3)° for mol­ecule *B*. In the crystal, two inter­molecular N—H⋯N hydrogen bonds lead to the formation of a dimer with *R*_2_^2^(10) graph-set notation. A Hirshfeld surface analysis revealed that H⋯H inter­actions are the most important inter­molecular inter­actions, contributing 40.9% to the Hirshfeld surface.

## Abstract 2

The crystal structure of the title compound comprises two independent mol­ecules that mainly differ in the orientation of the phenyl ring to the rest of the mol­ecule.

## Chemical context

Thio­amides and their derivatives are important representatives of organic compounds containing a sulfur atom. The presence of bifunctional properties in thio­amides, resulting from the presence of nitro­gen and sulfur atoms, and their participation in reactions as electrophilic or nucleophilic reagents can lead to the formation of different heterocyclic compounds. Several review articles have been published on the syntheses, physico-chemical properties and applications of thio­amides (Jagodziński, 2003 ▸; Belskaya *et al.*, 2010 ▸; Koketsu & Ishihara, 2007 ▸; Krayushkin *et al.*, 2004 ▸; Britsun *et al.*, 2008 ▸).

One of the methods of choice for the synthesis of widely used thio­amides is the Wilgerodt–Kindler reaction. As shown by previous studies, the Wilgerodt–Kindler reactions with 2-methyl­quinazoline-4-one went to the active methyl group in the position 2 and, accordingly, thio­amides were synthesized in a series of quinazoline derivatives (Shakhidoyatov *et al.*, 1997 ▸). Continuing our work in this direction, we have synthesized 2,3-di­methyl­quinazoline-4-one and studied the corresponding Wilgerodt–Kindler reactions.

During the reaction involving 2,3-di­methyl­quinazoline-4-one, sulfur, aniline, the solvent dimethyl sulfoxide and the catalyst sodium sulfide, the reaction went to the active methyl group in position 2 and new thio­amides of a number of deriv­atives of quinazoline-4-one were obtained. The synthesis and crystal structure of 3-methyl-4-oxo-*N*-phenyl-3,4-di­hydro­quinazoline-2-carbo­thio­amide, C_16_H_13_N_3_OS, is reported here. Relevant inter­molecular contacts were qu­anti­fied by using Hirshfeld surface analysis. 
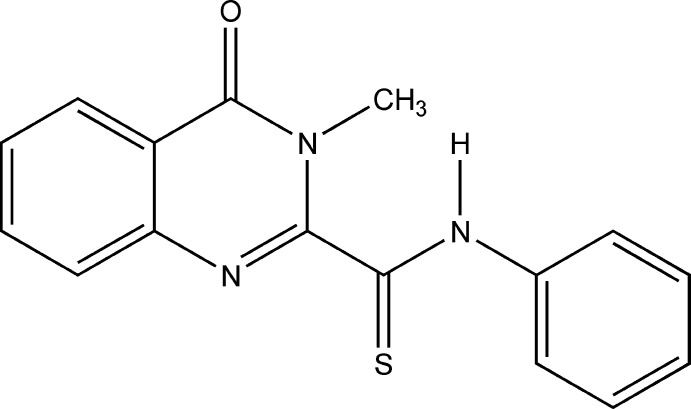


## Structural commentary

**Figure 1:**
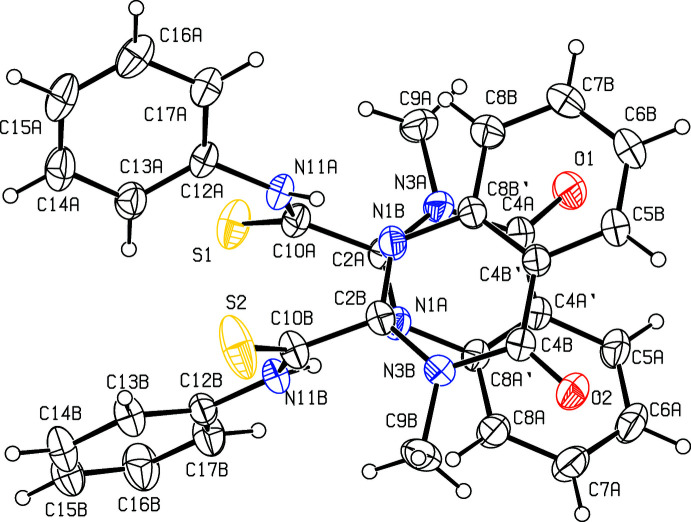
Asymmetric unit of the title compound with the atom-numbering scheme. Displacement ellipsoids for non-hydrogen atoms are drawn at the 30% probability level.

**Figure 2:**
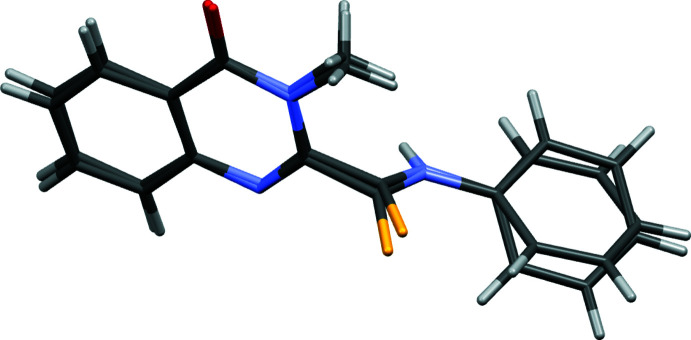
Overlay plot of the two independent mol­ecules in the title compound.

The title compound crystallizes with two mol­ecules, *A* and *B*, in the asymmetric unit (Fig. 1 ▸). In mol­ecules *A* and *B* the orientations of the quinazoline ring system and the phenyl ring relative to the thio­amide group differ, as shown by the values of the N3—C2—C10—S1 and C10—N11—C12—C13 torsion angles of 76.14 (19) and 49.3 (3)°, respectively, in mol­ecule *A* and 83.78 (19) and 5.4 (3)° in mol­ecule *B*. As a result, there are differences in the intra­molecular distances between the sulfur and hydrogen atoms in mol­ecules *A* and *B*. In mol­ecule *A*, the contacts S1*A*⋯H9*AB* and S1*A*⋯H13*A* are 2.873 and 2.897 Å whereas the corresponding distances in mol­ecule *B* are 3.054 and 2.578 Å. The phenyl and pyrimidine rings in both mol­ecules are essentially coplanar, with r.m.s. deviations of 0.0225 and 0.0119 Å for mol­ecule *A* and *B*, respectively. Fig. 2 ▸ shows that the pyrimidine moieties of the mol­ecules are almost superimposable.

## Supra­molecular features

**Figure 3:**
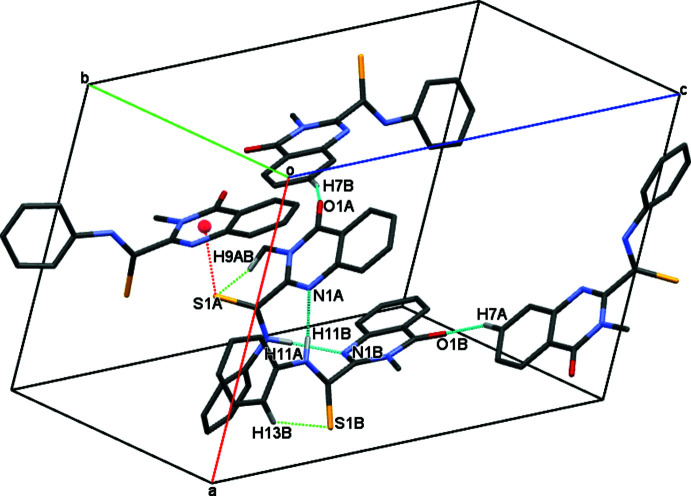
A diagram showing the intra­mol­ecular C—H⋯S (green dashed lines) and the inter­molecular N—H⋯N (light blue dashed lines) and C—H⋯O (blue dashed lines) hydrogen bonds, as well as C—S⋯π (red dashed lines) inter­actions present in the title compound. H atoms not involved in the inter­actions have been omitted for clarity.

**Figure 4:**
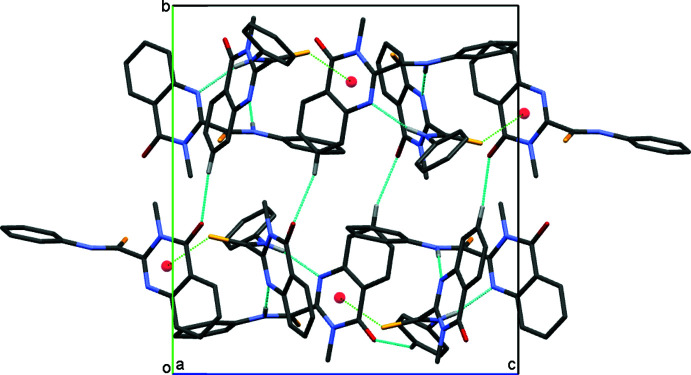
A view of the crystal packing of the title compound along the *a* axis. Inter­molecular hydrogen bonds and C—S⋯π inter­actions are displayed by blue and green dotted lines, respectively.

**Figure 5:**
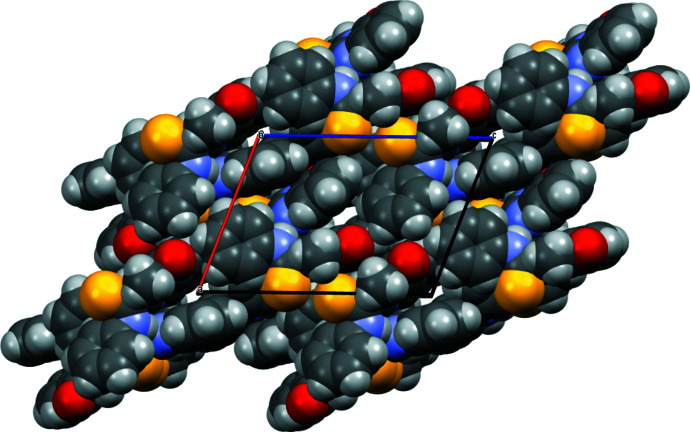
View of the narrow channels formed along the *b* axis.

In the crystal, mol­ecules *A* and *B* form a dimer with an 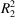 (10) ring motif through inter­mol­ecular N—H⋯N hydrogen bonds (Fig. 3 ▸, Table 1 ▸). In addition, mol­ecule *A* inter­acts with mol­ecule *B* by a C—H⋯ π inter­action (the C13*A*—H*⋯*Cg** 1 distance is 3.148 Å, *Cg*1 is the centroid of atoms C12*B*–C17*B*). Other weak C7*A*—H7*A*⋯O1*B*, C7*A*—H7*A* ⋯O1*B*, C7*B*—H7*B*⋯O1*A*, C9*A*—H9*AB*⋯S1*A* and C13*B*—H13*B*⋯S1*B* hydrogen bonds link adjacent dimers, forming supra­molecular layers expanding parallel to (010) (Fig. 4 ▸). The overall packing of mol­ecules leads to the formation of narrow channels along the *b-*axis direction, passing through nodes and the centre of the cell (Fig. 5 ▸).

## Hirshfeld surface analysis

**Figure 6:**
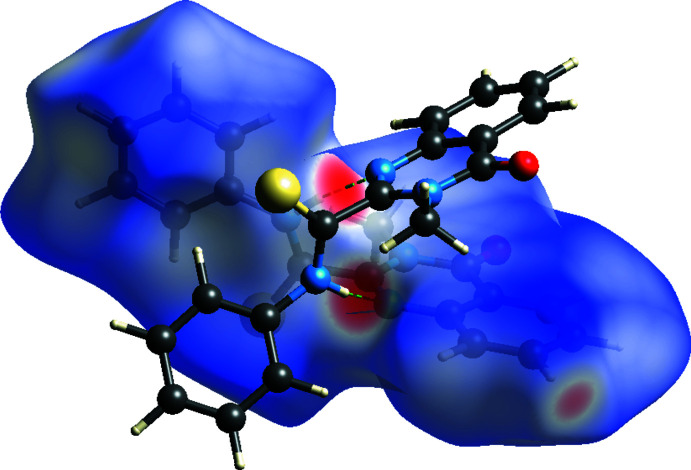
View of the three-dimensional Hirshfeld surface of the title compound plotted over *d*_norm_.

**Figure 7:**
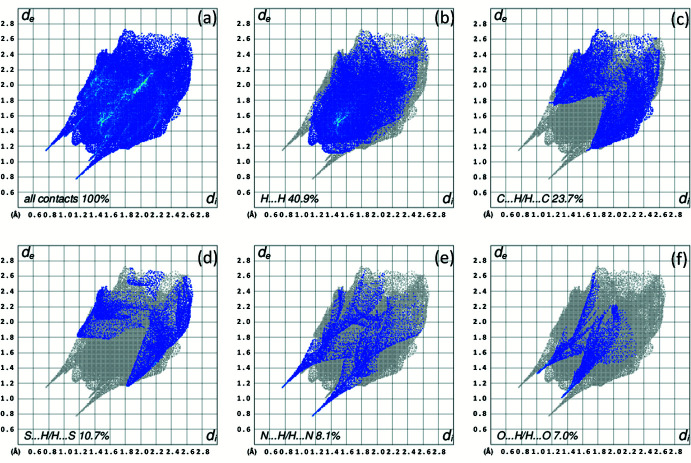
Two-dimensional fingerprint plots for the title compound, (*a*) for all contacts and delineated into (*b*) H⋯H, (*c*) C⋯H/H⋯C, (*d*) S⋯H/H⋯S, (*e*) N⋯H/H⋯N and (*f*) O⋯H/H⋯O contacts. *d*_i_ and *d*_e_ denote the closest inter­nal and external distances (in Å) from a point on the surface.

A Hirshfeld surface (HS) analysis (Spackman & Jayatilaka, 2009 ▸) was carried out using *CrystalExplorer17.5* (Turner *et al.*, 2017 ▸) to qu­antify and visualize inter­molecular inter­actions in the crystal structure of the title compound. The HS mapped with *d*_norm_ is represented in Fig. 6 ▸. The white surface indicates contacts with distances equal to the sum of van der Waals radii, and the red and blue colours indicate distances shorter or longer, respectively, than the van der Waals radii. The two-dimensional fingerprint plot for all contacts is depicted in Fig. 7 ▸ *a*, and delineated in H⋯H, C⋯H/H⋯C, S⋯H/H⋯S, N⋯H/H⋯N, and O⋯H/H⋯O contacts (Fig. 7 ▸ *b–f*) whereby H⋯H contacts are responsible for the largest contribution (40.9%) to the Hirshfeld surface. C⋯H/H⋯C contribute 23.7%, S⋯H/H⋯S contacts 10.7%, N⋯H/H⋯N contacts 8.1% and O⋯H/H⋯O contacts 7.0% to the total Hirshfeld surface. The contributions of further contacts are only minor and amount to C⋯C (4.0%), S⋯C/C⋯S (1.9%), N⋯C/C⋯N (1.2%), S⋯S (1.0%), S⋯C/C⋯S (0.6%), O⋯N/N⋯O (0.2%) and O⋯C/C⋯O (0.1%).

## Database survey

A search in the Cambridge Structural Database (CSD, version 5.41, update of January 2020; Groom *et al.*, 2016 ▸) revealed six matches for mol­ecules containing the 2,3-di­methyl­quinazolin-4(3*H*)-one moiety with a similar planar conformation as that in the title structure: AFOCIJ (Utayeva *et al.*, 2013 ▸), HOCYED (Voitenko *et al.*, 1999 ▸), MAHLOZ (Kotipalli *et al.*, 2016 ▸), MUDHIE (Baglai *et al.*, 2014 ▸), UTIDIM (Kundu *et al.*, 2016 ▸) and XODZIB (Saitkulov *et al.*, 2014 ▸). A search for the 2-methyl-*N*-phenyl­prop-2-ene­thio­amide moiety gave six hits: ADEKUQ (Xiao & Jian, 2006 ▸), AGECIB (Skelton & Massi, 2018 ▸), GOFFOY (Li *et al.*, 2014 ▸), GOXFUW (Li *et al.*, 2016 ▸), JURWEA (Guo *et al.*, 2015 ▸) and QAJVAY (Mereiter *et al.*, 2000 ▸).

## Synthesis and crystallization

0.435 g (0.0025 mol) of 2,3-di­methyl­quinazoline-4-one, 0.465 g (0.005 mol) of aniline, 0.24 g (0.0075 mol) of sulfur, 0.05 g of sodium sulfide (Na_2_S·9H_2_O) and 4 ml of dimethyl sulfoxide were injected into a round-bottomed flask with a volume of 100 ml. Then the reaction flask was heated to 403 K for 6 h. After the end of the reaction, the flask was cooled and 40 ml of an aqueous sodium hydroxide solution were added. The resulting mixture was filtered, then added to a dilute solution of sulfuric acid (pH 6). The formed precipitate was filtered off and recrystallized in methanol. In total, 0.5 g (64.0%) of the product were obtained, m.p. 481–483 K.

## Refinement

Crystal data, data collection and structure refinement details are summarized in Table 2 ▸. C-bound H atoms were positioned geometrically, with C—H = 0.96 Å (for methyl­ene H atoms) and C—H = 0.93 Å (for aromatic H atoms), and were refined with *U*_iso_(H) = 1.5*U*_eq_(C_meth­yl_) and 1.2*U*_eq_(C), respectively. H atoms bonded to nitro­gen were located in a difference-Fourier map, and their positional and isotropic displacement parameters were freely refined.

## Supplementary Material

Crystal structure: contains datablock(s) I. DOI: 10.1107/S2056989021013116/wm5629sup1.cif

Supporting information file. DOI: 10.1107/S2056989021013116/wm5629Isup3.cml

Structure factors: contains datablock(s) I. DOI: 10.1107/S2056989021013116/wm5629Isup3.hkl

CCDC reference: 2127513

Additional supporting information:  crystallographic information; 3D view; checkCIF report

## Figures and Tables

**Table 1:** Hydrogen-bond geometry (Å, °)

*D*—H⋯*A*	*D*—H	H⋯*A*	*D*⋯*A*	*D*—H⋯*A*
N1*A*—H11*A*⋯N1*B*	0.88 (2)	2.05 (2)	2.913 (2)	166.7 (18)
N1*B*—H11*B*⋯N1*A*	0.87 (2)	2.04 (2)	2.907 (2)	171.6 (19)
C9*A*—H9*AB*⋯S1*A*	0.96	2.87	3.424 (2)	118
C13*B*—H13*B*⋯S1*B*	0.93	2.58	3.243 (3)	129
C7*A* ^i^—H7*A*⋯O1*B*	0.93	2.49	3.386 (3)	162
C7*B* ^ii^—H7*B*⋯O1*A*	0.93	2.47	3.385 (3)	166

Symmetry codes: (i) -x+1, -y+2, -z+1; (ii) -x+1, -y+1, -z+1.

**Table 2:** Experimental details

Crystal data	
Chemical formula	C_16_H_13_N_3_OS
*M*_r_	295.35
Crystal system, space group	Monoclinic, *P*2_1_/*n*
Temperature (K)	566
*a*, *b*, *c* (Å)	11.7685 (3), 16.3641 (3), 16.3798 (3)
β (°)	110.646 (2)
*V* (Å^3^)	2951.85 (11)
*Z*	8
Radiation type	Cu *K*α
μ (mm^−1^)	1.96
Crystal size (mm)	0.25 × 0.23 × 0.20

Data collection	
Diffractometer	XtaLAB Synergy, Single source at home/near, HyPix3000
Absorption correction	Multi-scan (*CrysAlis PRO*; Rigaku OD, 2020 ▸)
*T*_min_, *T*_max_	0.639, 1.000
No. of measured, independent and observed [*I* > 2σ(*I*)] reflections	16801, 5685, 4788
*R*_int_	0.022
(sin θ/λ)_max_ (Å^−1^)	0.615

Refinement	
*R*[*F* ^2^ > 2σ(*F* ^2^)], *wR*(*F* ^2^), *S*	0.042, 0.121, 1.06
No. of reflections	5685
No. of parameters	390
H-atom treatment	H atoms treated by a mixture of independent and constrained refinement
Δρ_max_, Δρ_min_ (e Å^−3^)	0.32, −0.43

Computer programs: *CrysAlis PRO* (Rigaku OD, 2020 ▸), *SHELXT* (Sheldrick, 2015*a* ▸), *SHELXL* (Sheldrick, 2015*b* ▸), *PLATON* (Spek, 2020 ▸), *Mercury* (Macrae *et al.*, 2020 ▸) and *publCIF* (Westrip, 2010 ▸).

## supplementary crystallographic information

### Crystal data

**Table:**

C_16_H_13_N_3_OS	*F*(000) = 1232
*M**_r_* = 295.35	*D*_x_ = 1.329 Mg m^−^^3^
Monoclinic, *P*2_1_/*n*	Cu *K*α radiation, λ = 1.54184 Å
*a* = 11.7685 (3) Å	Cell parameters from 9141 reflections
*b* = 16.3641 (3) Å	θ = 2.7–71.1°
*c* = 16.3798 (3) Å	µ = 1.96 mm^−^^1^
β = 110.646 (2)°	*T* = 566 K
*V* = 2951.85 (11) Å^3^	Prismatic, yellow
*Z* = 8	0.25 × 0.23 × 0.20 mm

### Data collection

**Table:**

XtaLAB Synergy, Single source at home/near, HyPix3000 diffractometer	5685 independent reflections
Radiation source: micro-focus sealed X-ray tube	4788 reflections with *I* > 2σ(*I*)
Detector resolution: 10.00000 pixels mm^-1^	*R*_int_ = 0.022
ω scans	θ_max_ = 71.4°, θ_min_ = 4.0°
Absorption correction: multi-scan (CrysAlisPro; Rigaku OD, 2020)	*h* = −14→14
*T*_min_ = 0.639, *T*_max_ = 1.000	*k* = −19→17
16801 measured reflections	*l* = −19→20

### Refinement

**Table:**

Refinement on *F*^2^	Hydrogen site location: mixed
Least-squares matrix: full	H atoms treated by a mixture of independent and constrained refinement
*R*[*F*^2^ > 2σ(*F*^2^)] = 0.042	*w* = 1/[σ^2^(*F*_o_^2^) + (0.0602*P*)^2^ + 0.6163*P*] where *P* = (*F*_o_^2^ + 2*F*_c_^2^)/3
*wR*(*F*^2^) = 0.121	(Δ/σ)_max_ = 0.001
*S* = 1.06	Δρ_max_ = 0.32 e Å^−^^3^
5685 reflections	Δρ_min_ = −0.42 e Å^−^^3^
390 parameters	Extinction correction: SHELXL (Sheldrick, 2015a), Fc^*^=kFc[1+0.001xFc^2^λ^3^/sin(2θ)]^-1/4^
0 restraints	Extinction coefficient: 0.00124 (12)
Primary atom site location: structure-invariant direct methods	

### Special details

**Table:**

Geometry. All esds (except the esd in the dihedral angle between two l.s. planes) are estimated using the full covariance matrix. The cell esds are taken into account individually in the estimation of esds in distances, angles and torsion angles; correlations between esds in cell parameters are only used when they are defined by crystal symmetry. An approximate (isotropic) treatment of cell esds is used for estimating esds involving l.s. planes.

### Fractional atomic coordinates and isotropic or equivalent isotropic displacement parameters (Å^2^)

**Table:**

	*x*	*y*	*z*	*U*_iso_*/*U*_eq_	
S1A	0.46771 (5)	0.62649 (4)	0.89046 (3)	0.07206 (19)	
O1A	0.69766 (15)	0.59324 (9)	0.65155 (11)	0.0755 (4)	
N1A	0.48752 (12)	0.75527 (8)	0.72159 (9)	0.0456 (3)	
N1B	0.22774 (13)	0.73699 (9)	0.57212 (9)	0.0474 (3)	
S1B	0.04219 (5)	0.86597 (6)	0.63642 (4)	0.1030 (3)	
O1B	0.30121 (15)	0.90703 (8)	0.41700 (10)	0.0690 (4)	
C2A	0.49005 (14)	0.67801 (10)	0.73853 (10)	0.0430 (4)	
C2B	0.22279 (15)	0.81489 (10)	0.58376 (10)	0.0444 (4)	
N3A	0.55988 (13)	0.62176 (8)	0.71623 (10)	0.0487 (3)	
N3B	0.24924 (13)	0.87402 (8)	0.53417 (9)	0.0461 (3)	
C4A	0.63788 (17)	0.64470 (11)	0.67217 (12)	0.0523 (4)	
C4B	0.28038 (16)	0.85368 (11)	0.46179 (11)	0.0476 (4)	
C4A'	0.63881 (16)	0.73161 (11)	0.65427 (12)	0.0488 (4)	
C4B'	0.28702 (15)	0.76640 (10)	0.44719 (10)	0.0441 (4)	
C5A	0.7150 (2)	0.76334 (13)	0.61289 (15)	0.0663 (5)	
H5A	0.767186	0.728822	0.597752	0.080*	
C5B	0.31931 (18)	0.73805 (12)	0.37776 (12)	0.0572 (5)	
H5B	0.336243	0.775028	0.340470	0.069*	
C6A	0.7128 (2)	0.84521 (14)	0.59469 (16)	0.0719 (6)	
H6A	0.763410	0.866124	0.567158	0.086*	
C6B	0.3260 (2)	0.65590 (14)	0.36481 (14)	0.0699 (6)	
H6B	0.347755	0.637057	0.318813	0.084*	
C7A	0.6353 (2)	0.89685 (13)	0.61719 (15)	0.0669 (5)	
H7A	0.633301	0.952178	0.603750	0.080*	
C7B	0.3002 (2)	0.60060 (13)	0.42011 (16)	0.0738 (6)	
H7B	0.304581	0.544836	0.410662	0.089*	
C8A	0.56154 (18)	0.86710 (11)	0.65916 (14)	0.0570 (5)	
H8A	0.510698	0.902433	0.674732	0.068*	
C8B	0.2684 (2)	0.62723 (12)	0.48872 (14)	0.0644 (5)	
H8B	0.251967	0.589664	0.525684	0.077*	
C8A'	0.56261 (15)	0.78398 (10)	0.67853 (11)	0.0443 (4)	
C8B'	0.26094 (16)	0.71106 (10)	0.50270 (11)	0.0455 (4)	
C9A	0.5550 (2)	0.53429 (12)	0.73431 (16)	0.0712 (6)	
H9AA	0.543485	0.503622	0.682028	0.107*	
H9AB	0.488631	0.524014	0.754066	0.107*	
H9AC	0.629728	0.517988	0.778655	0.107*	
C9B	0.2411 (2)	0.96167 (12)	0.55107 (14)	0.0673 (5)	
H9BA	0.308740	0.989779	0.544197	0.101*	
H9BB	0.242392	0.969168	0.609523	0.101*	
H9BC	0.166757	0.983288	0.510518	0.101*	
C10A	0.40897 (15)	0.64886 (10)	0.78644 (11)	0.0464 (4)	
C10B	0.18531 (16)	0.84207 (12)	0.65864 (11)	0.0520 (4)	
N11A	0.29256 (13)	0.64598 (9)	0.73505 (9)	0.0459 (3)	
N11B	0.27899 (14)	0.84235 (9)	0.73404 (9)	0.0458 (3)	
H11A	0.2709 (18)	0.6657 (12)	0.6816 (13)	0.056 (5)*	
H11B	0.3453 (19)	0.8211 (12)	0.7308 (13)	0.060 (6)*	
C12A	0.19183 (15)	0.62418 (10)	0.76017 (11)	0.0458 (4)	
C12B	0.28641 (16)	0.86210 (10)	0.82016 (10)	0.0460 (4)	
C13A	0.17430 (18)	0.65831 (12)	0.83199 (13)	0.0579 (5)	
H13A	0.231162	0.694273	0.868105	0.070*	
C13B	0.1889 (2)	0.88031 (13)	0.84467 (13)	0.0615 (5)	
H13B	0.110617	0.880890	0.803547	0.074*	
C14A	0.0710 (2)	0.63823 (14)	0.84934 (16)	0.0687 (6)	
H14A	0.058768	0.660791	0.897690	0.082*	
C14B	0.2088 (2)	0.89785 (15)	0.93166 (14)	0.0733 (6)	
H14B	0.143062	0.910500	0.948369	0.088*	
C15A	−0.01364 (19)	0.58550 (15)	0.79631 (16)	0.0728 (6)	
H15A	−0.083359	0.573110	0.808191	0.087*	
C15B	0.3230 (2)	0.89684 (15)	0.99301 (13)	0.0743 (6)	
H15B	0.335113	0.909440	1.050847	0.089*	
C16A	0.00499 (18)	0.55107 (15)	0.72556 (15)	0.0713 (6)	
H16A	−0.051928	0.514896	0.689846	0.086*	
C16B	0.4194 (2)	0.87709 (17)	0.96852 (14)	0.0799 (7)	
H16B	0.497078	0.875101	1.010346	0.096*	
C17A	0.10845 (17)	0.57004 (13)	0.70712 (12)	0.0580 (5)	
H17A	0.121368	0.546455	0.659469	0.070*	
C17B	0.40270 (19)	0.86009 (14)	0.88256 (12)	0.0637 (5)	
H17B	0.468896	0.847365	0.866451	0.076*	

### Atomic displacement parameters (Å^2^)

**Table:**

	*U*^11^	*U*^22^	*U*^33^	*U*^12^	*U*^13^	*U*^23^
S1A	0.0501 (3)	0.1132 (5)	0.0526 (3)	−0.0013 (3)	0.0178 (2)	0.0220 (3)
O1A	0.0852 (10)	0.0582 (8)	0.1095 (12)	0.0080 (7)	0.0668 (10)	−0.0077 (8)
N1A	0.0427 (7)	0.0479 (8)	0.0540 (8)	0.0015 (6)	0.0267 (6)	0.0025 (6)
N1B	0.0546 (8)	0.0508 (8)	0.0435 (7)	0.0033 (6)	0.0257 (6)	0.0007 (6)
S1B	0.0536 (3)	0.1936 (8)	0.0591 (3)	0.0382 (4)	0.0165 (3)	−0.0226 (4)
O1B	0.0907 (10)	0.0565 (8)	0.0774 (9)	−0.0040 (7)	0.0513 (8)	0.0100 (7)
C2A	0.0387 (8)	0.0476 (9)	0.0469 (8)	−0.0015 (6)	0.0201 (7)	0.0002 (7)
C2B	0.0423 (9)	0.0539 (10)	0.0380 (8)	0.0038 (7)	0.0152 (7)	−0.0023 (7)
N3A	0.0499 (8)	0.0422 (7)	0.0625 (9)	−0.0013 (6)	0.0304 (7)	−0.0013 (6)
N3B	0.0495 (8)	0.0443 (7)	0.0464 (7)	−0.0005 (6)	0.0195 (6)	−0.0044 (6)
C4A	0.0521 (10)	0.0519 (10)	0.0633 (11)	−0.0020 (8)	0.0333 (9)	−0.0068 (8)
C4B	0.0489 (9)	0.0503 (9)	0.0484 (9)	−0.0023 (7)	0.0229 (8)	0.0002 (7)
C4A'	0.0469 (9)	0.0512 (9)	0.0569 (10)	−0.0034 (7)	0.0290 (8)	−0.0030 (7)
C4B'	0.0445 (9)	0.0489 (9)	0.0425 (8)	−0.0018 (7)	0.0198 (7)	−0.0030 (7)
C5A	0.0646 (12)	0.0689 (13)	0.0855 (14)	−0.0023 (10)	0.0513 (11)	0.0005 (10)
C5B	0.0658 (12)	0.0649 (11)	0.0512 (10)	0.0001 (9)	0.0335 (9)	−0.0045 (8)
C6A	0.0704 (14)	0.0738 (14)	0.0899 (15)	−0.0128 (11)	0.0511 (12)	0.0088 (11)
C6B	0.0848 (15)	0.0734 (13)	0.0648 (12)	0.0046 (11)	0.0429 (11)	−0.0170 (10)
C7A	0.0699 (13)	0.0544 (11)	0.0843 (14)	−0.0078 (10)	0.0371 (11)	0.0123 (10)
C7B	0.0952 (17)	0.0519 (11)	0.0867 (15)	0.0033 (11)	0.0476 (13)	−0.0166 (10)
C8A	0.0580 (11)	0.0485 (10)	0.0733 (12)	0.0009 (8)	0.0339 (10)	0.0057 (8)
C8B	0.0857 (15)	0.0480 (10)	0.0714 (13)	0.0014 (9)	0.0425 (11)	−0.0013 (8)
C8A'	0.0409 (8)	0.0473 (9)	0.0498 (9)	−0.0028 (7)	0.0222 (7)	0.0001 (7)
C8B'	0.0504 (9)	0.0468 (9)	0.0442 (8)	0.0017 (7)	0.0227 (7)	−0.0018 (7)
C9A	0.0849 (15)	0.0444 (10)	0.0987 (16)	−0.0026 (10)	0.0505 (13)	0.0025 (10)
C9B	0.0868 (15)	0.0460 (10)	0.0687 (12)	0.0036 (10)	0.0270 (11)	−0.0114 (9)
C10A	0.0412 (9)	0.0513 (9)	0.0518 (9)	−0.0005 (7)	0.0228 (7)	0.0051 (7)
C10B	0.0501 (10)	0.0655 (11)	0.0435 (9)	0.0082 (8)	0.0203 (8)	−0.0063 (8)
N11A	0.0415 (7)	0.0561 (8)	0.0453 (8)	−0.0012 (6)	0.0217 (6)	0.0100 (6)
N11B	0.0475 (8)	0.0537 (8)	0.0415 (7)	0.0071 (6)	0.0222 (6)	−0.0045 (6)
C12A	0.0385 (8)	0.0533 (9)	0.0500 (9)	0.0032 (7)	0.0213 (7)	0.0141 (7)
C12B	0.0560 (10)	0.0462 (9)	0.0415 (8)	0.0035 (7)	0.0241 (8)	−0.0018 (6)
C13A	0.0570 (11)	0.0587 (11)	0.0684 (11)	0.0054 (9)	0.0348 (9)	0.0052 (9)
C13B	0.0619 (12)	0.0770 (13)	0.0520 (10)	0.0144 (10)	0.0280 (9)	−0.0044 (9)
C14A	0.0647 (13)	0.0808 (14)	0.0776 (14)	0.0150 (11)	0.0461 (12)	0.0154 (11)
C14B	0.0842 (16)	0.0910 (15)	0.0588 (12)	0.0145 (12)	0.0426 (12)	−0.0071 (11)
C15A	0.0501 (11)	0.0950 (16)	0.0849 (15)	0.0072 (11)	0.0380 (11)	0.0303 (13)
C15B	0.0956 (17)	0.0875 (15)	0.0461 (10)	−0.0038 (13)	0.0329 (11)	−0.0109 (10)
C16A	0.0486 (11)	0.0888 (15)	0.0742 (13)	−0.0137 (10)	0.0187 (10)	0.0193 (11)
C16B	0.0736 (15)	0.116 (2)	0.0473 (11)	−0.0050 (13)	0.0174 (10)	−0.0126 (11)
C17A	0.0507 (10)	0.0729 (12)	0.0521 (10)	−0.0069 (9)	0.0203 (8)	0.0098 (9)
C17B	0.0556 (11)	0.0883 (15)	0.0490 (10)	0.0010 (10)	0.0207 (9)	−0.0083 (9)

### Geometric parameters (Å, º)

**Table:**

S1A—C10A	1.6385 (17)	C8B—C8B'	1.399 (2)
O1A—C4A	1.219 (2)	C8B—H8B	0.9300
N1A—C2A	1.292 (2)	C9A—H9AA	0.9600
N1A—C8A'	1.392 (2)	C9A—H9AB	0.9600
N1B—C2B	1.293 (2)	C9A—H9AC	0.9600
N1B—C8B'	1.392 (2)	C9B—H9BA	0.9600
S1B—C10B	1.6401 (18)	C9B—H9BB	0.9600
O1B—C4B	1.220 (2)	C9B—H9BC	0.9600
C2A—N3A	1.367 (2)	C10A—N11A	1.332 (2)
C2A—C10A	1.511 (2)	C10B—N11B	1.334 (2)
C2B—N3B	1.368 (2)	N11A—C12A	1.430 (2)
C2B—C10B	1.510 (2)	N11A—H11A	0.88 (2)
N3A—C4A	1.404 (2)	N11B—C12B	1.420 (2)
N3A—C9A	1.467 (2)	N11B—H11B	0.87 (2)
N3B—C4B	1.399 (2)	C12A—C17A	1.379 (3)
N3B—C9B	1.470 (2)	C12A—C13A	1.382 (3)
C4A—C4A'	1.453 (3)	C12B—C13B	1.375 (2)
C4B—C4B'	1.455 (2)	C12B—C17B	1.390 (3)
C4A'—C8A'	1.396 (2)	C13A—C14A	1.382 (3)
C4A'—C5A	1.401 (2)	C13A—H13A	0.9300
C4B'—C8B'	1.392 (2)	C13B—C14B	1.390 (3)
C4B'—C5B	1.399 (2)	C13B—H13B	0.9300
C5A—C6A	1.371 (3)	C14A—C15A	1.372 (3)
C5A—H5A	0.9300	C14A—H14A	0.9300
C5B—C6B	1.367 (3)	C14B—C15B	1.365 (3)
C5B—H5B	0.9300	C14B—H14B	0.9300
C6A—C7A	1.385 (3)	C15A—C16A	1.374 (3)
C6A—H6A	0.9300	C15A—H15A	0.9300
C6B—C7B	1.387 (3)	C15B—C16B	1.369 (3)
C6B—H6B	0.9300	C15B—H15B	0.9300
C7A—C8A	1.373 (3)	C16A—C17A	1.389 (3)
C7A—H7A	0.9300	C16A—H16A	0.9300
C7B—C8B	1.375 (3)	C16B—C17B	1.380 (3)
C7B—H7B	0.9300	C16B—H16B	0.9300
C8A—C8A'	1.396 (2)	C17A—H17A	0.9300
C8A—H8A	0.9300	C17B—H17B	0.9300
			
C2A—N1A—C8A'	117.91 (14)	H9AA—C9A—H9AB	109.5
C2B—N1B—C8B'	117.44 (14)	N3A—C9A—H9AC	109.5
N1A—C2A—N3A	124.89 (14)	H9AA—C9A—H9AC	109.5
N1A—C2A—C10A	116.71 (14)	H9AB—C9A—H9AC	109.5
N3A—C2A—C10A	118.39 (14)	N3B—C9B—H9BA	109.5
N1B—C2B—N3B	125.33 (14)	N3B—C9B—H9BB	109.5
N1B—C2B—C10B	116.84 (15)	H9BA—C9B—H9BB	109.5
N3B—C2B—C10B	117.83 (15)	N3B—C9B—H9BC	109.5
C2A—N3A—C4A	121.39 (14)	H9BA—C9B—H9BC	109.5
C2A—N3A—C9A	122.23 (15)	H9BB—C9B—H9BC	109.5
C4A—N3A—C9A	116.35 (15)	N11A—C10A—C2A	112.34 (14)
C2B—N3B—C4B	121.15 (14)	N11A—C10A—S1A	127.74 (13)
C2B—N3B—C9B	122.32 (15)	C2A—C10A—S1A	119.91 (12)
C4B—N3B—C9B	116.47 (15)	N11B—C10B—C2B	111.73 (14)
O1A—C4A—N3A	120.23 (17)	N11B—C10B—S1B	130.79 (13)
O1A—C4A—C4A'	125.05 (17)	C2B—C10B—S1B	117.47 (13)
N3A—C4A—C4A'	114.72 (14)	C10A—N11A—C12A	126.80 (14)
O1B—C4B—N3B	120.52 (16)	C10A—N11A—H11A	119.1 (13)
O1B—C4B—C4B'	124.74 (16)	C12A—N11A—H11A	113.5 (13)
N3B—C4B—C4B'	114.73 (14)	C10B—N11B—C12B	131.43 (15)
C8A'—C4A'—C5A	119.71 (17)	C10B—N11B—H11B	114.1 (13)
C8A'—C4A'—C4A	119.49 (15)	C12B—N11B—H11B	114.0 (13)
C5A—C4A'—C4A	120.80 (16)	C17A—C12A—C13A	120.63 (17)
C8B'—C4B'—C5B	120.06 (16)	C17A—C12A—N11A	117.17 (16)
C8B'—C4B'—C4B	119.62 (14)	C13A—C12A—N11A	122.12 (17)
C5B—C4B'—C4B	120.33 (16)	C13B—C12B—C17B	119.80 (16)
C6A—C5A—C4A'	120.07 (19)	C13B—C12B—N11B	125.03 (17)
C6A—C5A—H5A	120.0	C17B—C12B—N11B	115.15 (16)
C4A'—C5A—H5A	120.0	C14A—C13A—C12A	119.0 (2)
C6B—C5B—C4B'	119.92 (18)	C14A—C13A—H13A	120.5
C6B—C5B—H5B	120.0	C12A—C13A—H13A	120.5
C4B'—C5B—H5B	120.0	C12B—C13B—C14B	119.2 (2)
C5A—C6A—C7A	120.17 (18)	C12B—C13B—H13B	120.4
C5A—C6A—H6A	119.9	C14B—C13B—H13B	120.4
C7A—C6A—H6A	119.9	C15A—C14A—C13A	121.0 (2)
C5B—C6B—C7B	120.16 (18)	C15A—C14A—H14A	119.5
C5B—C6B—H6B	119.9	C13A—C14A—H14A	119.5
C7B—C6B—H6B	119.9	C15B—C14B—C13B	121.2 (2)
C8A—C7A—C6A	120.58 (19)	C15B—C14B—H14B	119.4
C8A—C7A—H7A	119.7	C13B—C14B—H14B	119.4
C6A—C7A—H7A	119.7	C14A—C15A—C16A	119.72 (19)
C8B—C7B—C6B	120.80 (19)	C14A—C15A—H15A	120.1
C8B—C7B—H7B	119.6	C16A—C15A—H15A	120.1
C6B—C7B—H7B	119.6	C14B—C15B—C16B	119.32 (19)
C7A—C8A—C8A'	120.18 (18)	C14B—C15B—H15B	120.3
C7A—C8A—H8A	119.9	C16B—C15B—H15B	120.3
C8A'—C8A—H8A	119.9	C15A—C16A—C17A	120.3 (2)
C7B—C8B—C8B'	119.73 (19)	C15A—C16A—H16A	119.9
C7B—C8B—H8B	120.1	C17A—C16A—H16A	119.9
C8B'—C8B—H8B	120.1	C15B—C16B—C17B	120.8 (2)
N1A—C8A'—C4A'	121.56 (15)	C15B—C16B—H16B	119.6
N1A—C8A'—C8A	119.17 (15)	C17B—C16B—H16B	119.6
C4A'—C8A'—C8A	119.26 (16)	C12A—C17A—C16A	119.35 (19)
C4B'—C8B'—N1B	121.68 (15)	C12A—C17A—H17A	120.3
C4B'—C8B'—C8B	119.33 (16)	C16A—C17A—H17A	120.3
N1B—C8B'—C8B	118.99 (16)	C16B—C17B—C12B	119.6 (2)
N3A—C9A—H9AA	109.5	C16B—C17B—H17B	120.2
N3A—C9A—H9AB	109.5	C12B—C17B—H17B	120.2
			
C8A'—N1A—C2A—N3A	−1.0 (3)	C4A—C4A'—C8A'—C8A	−178.35 (17)
C8A'—N1A—C2A—C10A	179.14 (14)	C7A—C8A—C8A'—N1A	179.63 (18)
C8B'—N1B—C2B—N3B	−1.0 (3)	C7A—C8A—C8A'—C4A'	−0.4 (3)
C8B'—N1B—C2B—C10B	179.33 (14)	C5B—C4B'—C8B'—N1B	−179.59 (16)
N1A—C2A—N3A—C4A	0.6 (3)	C4B—C4B'—C8B'—N1B	0.4 (3)
C10A—C2A—N3A—C4A	−179.57 (16)	C5B—C4B'—C8B'—C8B	0.6 (3)
N1A—C2A—N3A—C9A	−177.51 (18)	C4B—C4B'—C8B'—C8B	−179.50 (18)
C10A—C2A—N3A—C9A	2.4 (3)	C2B—N1B—C8B'—C4B'	−0.5 (2)
N1B—C2B—N3B—C4B	2.7 (3)	C2B—N1B—C8B'—C8B	179.35 (18)
C10B—C2B—N3B—C4B	−177.67 (15)	C7B—C8B—C8B'—C4B'	−0.6 (3)
N1B—C2B—N3B—C9B	179.73 (17)	C7B—C8B—C8B'—N1B	179.5 (2)
C10B—C2B—N3B—C9B	−0.6 (2)	N1A—C2A—C10A—N11A	75.1 (2)
C2A—N3A—C4A—O1A	−178.66 (18)	N3A—C2A—C10A—N11A	−104.80 (18)
C9A—N3A—C4A—O1A	−0.5 (3)	N1A—C2A—C10A—S1A	−103.97 (17)
C2A—N3A—C4A—C4A'	0.9 (3)	N3A—C2A—C10A—S1A	76.14 (19)
C9A—N3A—C4A—C4A'	179.10 (17)	N1B—C2B—C10B—N11B	83.6 (2)
C2B—N3B—C4B—O1B	178.41 (17)	N3B—C2B—C10B—N11B	−96.12 (19)
C9B—N3B—C4B—O1B	1.2 (3)	N1B—C2B—C10B—S1B	−96.53 (18)
C2B—N3B—C4B—C4B'	−2.6 (2)	N3B—C2B—C10B—S1B	83.78 (19)
C9B—N3B—C4B—C4B'	−179.78 (16)	C2A—C10A—N11A—C12A	−177.47 (15)
O1A—C4A—C4A'—C8A'	177.6 (2)	S1A—C10A—N11A—C12A	1.5 (3)
N3A—C4A—C4A'—C8A'	−1.9 (3)	C2B—C10B—N11B—C12B	−179.09 (17)
O1A—C4A—C4A'—C5A	−2.2 (3)	S1B—C10B—N11B—C12B	1.0 (3)
N3A—C4A—C4A'—C5A	178.19 (18)	C10A—N11A—C12A—C17A	−133.70 (19)
O1B—C4B—C4B'—C8B'	−179.87 (18)	C10A—N11A—C12A—C13A	49.3 (3)
N3B—C4B—C4B'—C8B'	1.1 (2)	C10B—N11B—C12B—C13B	5.4 (3)
O1B—C4B—C4B'—C5B	0.1 (3)	C10B—N11B—C12B—C17B	−176.2 (2)
N3B—C4B—C4B'—C5B	−178.91 (16)	C17A—C12A—C13A—C14A	−0.8 (3)
C8A'—C4A'—C5A—C6A	−1.4 (3)	N11A—C12A—C13A—C14A	176.03 (17)
C4A—C4A'—C5A—C6A	178.5 (2)	C17B—C12B—C13B—C14B	1.1 (3)
C8B'—C4B'—C5B—C6B	−0.4 (3)	N11B—C12B—C13B—C14B	179.36 (19)
C4B—C4B'—C5B—C6B	179.66 (18)	C12A—C13A—C14A—C15A	−0.3 (3)
C4A'—C5A—C6A—C7A	0.1 (4)	C12B—C13B—C14B—C15B	−0.4 (4)
C4B'—C5B—C6B—C7B	0.3 (3)	C13A—C14A—C15A—C16A	1.0 (3)
C5A—C6A—C7A—C8A	1.1 (4)	C13B—C14B—C15B—C16B	−0.9 (4)
C5B—C6B—C7B—C8B	−0.3 (4)	C14A—C15A—C16A—C17A	−0.6 (3)
C6A—C7A—C8A—C8A'	−0.9 (3)	C14B—C15B—C16B—C17B	1.5 (4)
C6B—C7B—C8B—C8B'	0.5 (4)	C13A—C12A—C17A—C16A	1.2 (3)
C2A—N1A—C8A'—C4A'	−0.1 (2)	N11A—C12A—C17A—C16A	−175.84 (17)
C2A—N1A—C8A'—C8A	179.82 (16)	C15A—C16A—C17A—C12A	−0.4 (3)
C5A—C4A'—C8A'—N1A	−178.49 (18)	C15B—C16B—C17B—C12B	−0.8 (4)
C4A—C4A'—C8A'—N1A	1.6 (3)	C13B—C12B—C17B—C16B	−0.5 (3)
C5A—C4A'—C8A'—C8A	1.5 (3)	N11B—C12B—C17B—C16B	−179.0 (2)

### Hydrogen-bond geometry (Å, º)

**Table:**

*D*—H···*A*	*D*—H	H···*A*	*D*···*A*	*D*—H···*A*
N1*A*—H11*A*···N1*B*	0.88 (2)	2.05 (2)	2.913 (2)	166.7 (18)
N1*B*—H11*B*···N1*A*	0.87 (2)	2.04 (2)	2.907 (2)	171.6 (19)
C9*A*—H9*AB*···S1*A*	0.96	2.87	3.424 (2)	118
C13*B*—H13*B*···S1*B*	0.93	2.58	3.243 (3)	129
C7*A*^i^—H7*A*···O1*B*	0.93	2.49	3.386 (3)	162
C7*B*^ii^—H7*B*···O1*A*	0.93	2.47	3.385 (3)	166

Symmetry codes: (i) −*x*+1, −*y*+2, −*z*+1; (ii) −*x*+1, −*y*+1, −*z*+1.
